# Cellular Composition of the Preoptic Area Regulating Sleep, Parental, and Sexual Behavior

**DOI:** 10.3389/fnins.2021.649159

**Published:** 2021-03-31

**Authors:** Yousuke Tsuneoka, Hiromasa Funato

**Affiliations:** ^1^Department of Anatomy, Faculty of Medicine, Toho University, Tokyo, Japan; ^2^International Institute for Integrative Sleep Medicine (WPI-IIIS), University of Tsukuba, Ibaraki, Japan

**Keywords:** preoptic area, parental behavior, male sexual behavior, sexual dimorphism, galanin, Moxd1, NREM sleep, REM sleep

## Abstract

The preoptic area (POA) has long been recognized as a sleep center, first proposed by von Economo. The POA, especially the medial POA (MPOA), is also involved in the regulation of various innate functions such as sexual and parental behaviors. Consistent with its many roles, the MPOA is composed of subregions that are identified by different gene and protein expressions. This review addresses the current understanding of the molecular and cellular architecture of POA neurons in relation to sleep and reproductive behavior. Optogenetic and pharmacogenetic studies have revealed a diverse group of neurons within the POA that exhibit different neural activity patterns depending on vigilance states and whose activity can enhance or suppress wake, non-rapid eye movement (NREM) sleep, or rapid eye movement (REM) sleep. These sleep-regulating neurons are not restricted to the ventrolateral POA (VLPO) region but are widespread in the lateral MPOA and LPOA as well. Neurons expressing galanin also express gonadal steroid receptors and regulate motivational aspects of reproductive behaviors. Moxd1, a novel marker of sexually dimorphic nuclei (SDN), visualizes the SDN of the POA (SDN-POA). The role of the POA in sleep and other innate behaviors has been addressed separately; more integrated observation will be necessary to obtain physiologically relevant insight that penetrates the different dimensions of animal behavior.

## Introduction

The preoptic area (POA), the most anterior part of the hypothalamus, is a brain region that has a complex structure consisting of different groups of neurons that control various functions and behaviors essential for the survival of individuals and species. The POA, especially the medial POA (MPOA), plays a crucial role in the regulation of sleep and reproduction-associated behavior, such as parenting and male mating (Tsuneoka et al., 2013; Hull and Dominguez, 2015; Tan et al., 2016; Chung et al., 2017; Kohl et al., 2018). The MPOA is also involved in aggression, predation, feeding, and body temperature regulation (Hashikawa et al., 2016; Han et al., 2017; Ishii et al., 2017; Park et al., 2018; Morrison and Nakamura, 2019). Importantly, some of the MPOA nuclei show distinct sexual dimorphism (Simerly et al., 1984; Bloch and Gorski, 1988; Alexander et al., 1991; Orikasa and Sakuma, 2010; Tsuneoka et al., 2017a). The POA’s various important roles attracted researchers, but at the same time, many researchers believe that the POA is complex and difficult to understand. One of the reasons why there is so much confusion surrounding the POA is the inconsistent nomenclature of its subregions/nuclei. Even the two most commonly used mouse brain atlases are not consistent regarding the terminology and subnucleus structure of the POA (Paxinos et al., 2012; Allen Brain Atlas^1^). Therefore, in this review, we first describe the organization of the POA and then discuss how this region regulates sleep/wakefulness and various other behaviors.

## Cellular Organization and Gene Expression in the POA

**FIGURE 1:**
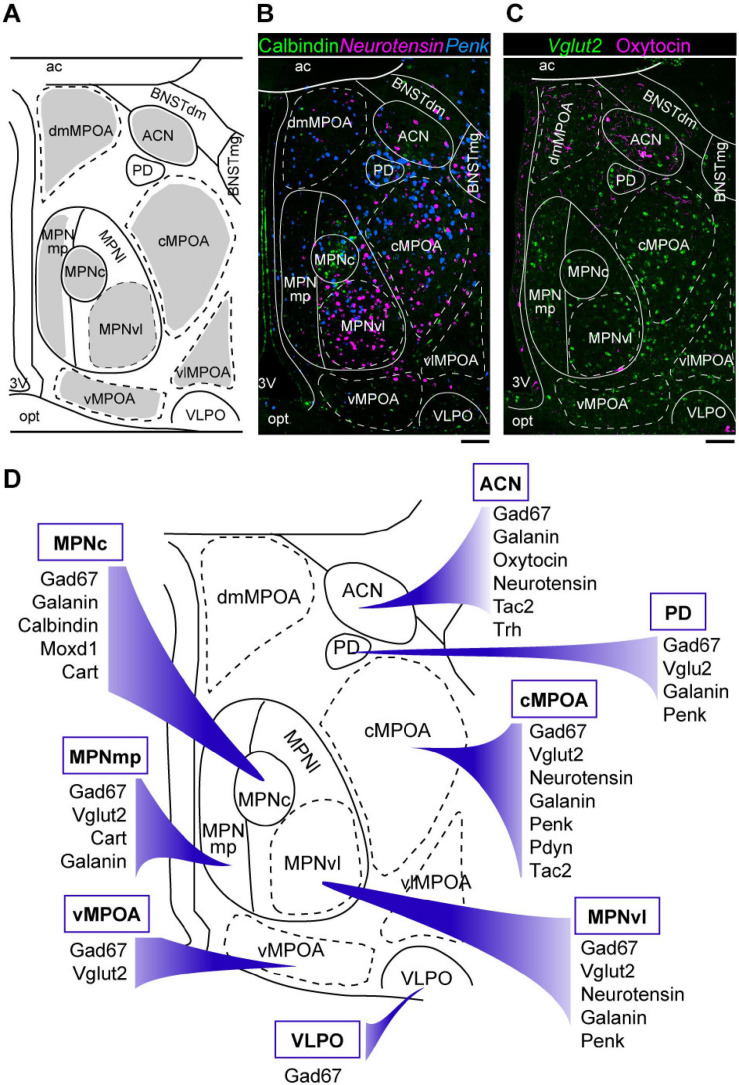
Neuroanatomy and heterogeneity of the medial preoptic area. **(A)** Coronal mouse brain diagram showing the MPOA subregions at the level of the MPNc (bregma, –0.02 mm). **(B)** Double ISH for *Penk* (blue) and *neurotensin* (magenta) mRNAs with immunostaining for calbindin (green). **(C)** ISH for *Vglut2* (green) mRNA with immunostaining for oxytocin (magenta). **(D)** Summary of marker genes expression in selected MPOA subregions. Each subregion has a characteristic pattern of marker expression. Scale bars: 100 μm. 3v, third ventricle; ac, anterior commissure; ACN, anterior commissural nucleus; BNSTdm, dorsomedial nucleus of the BNST; BNSTmg, magnocellular nucleus of the BNST; cMPOA, central part of the MPOA; dmMPOA, dorsomedial part of the MPOA; MPNc, central part of the MPN; MPNl, lateral part of the MPN; MPNmp, posteromedial part of the MPN; MPNvl, ventrolateral part of the MPN; opt, optic tract; PD, posterodorsal preoptic nucleus; vMPOA, ventral part of the MPOA; vlMPOA, ventrolateral part of the MPOA; VLPO, ventrolateral preoptic nucleus. Modified from Tsuneoka et al. (2017b).

**FIGURE 2:**
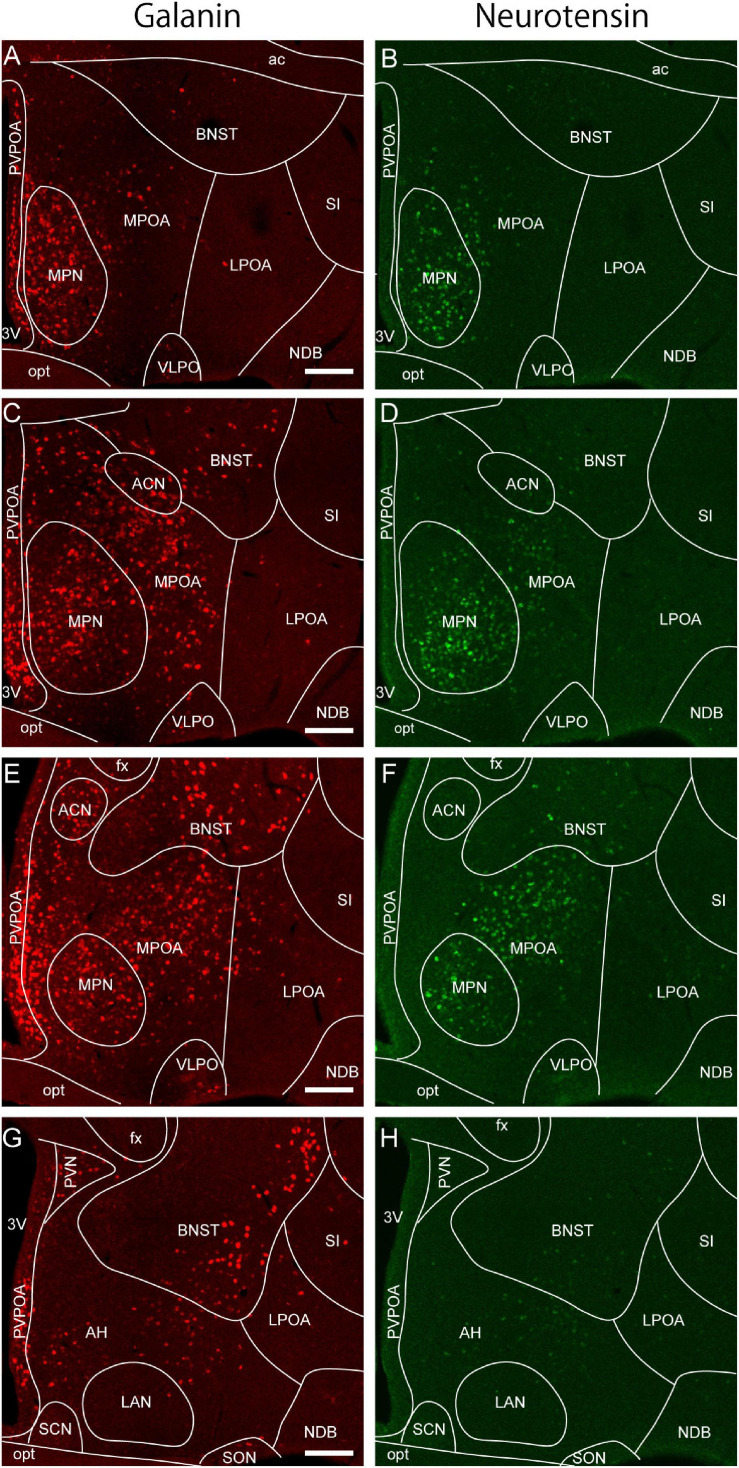
*Galanin* and *neurotensin* mRNA expression in the preoptic area. **(A,B)** Double ISH for **(A)** *galanin* and **(B)** *neurotensin* of a coronal section of the mouse brain (bregma + 0.10 mm). **(C,D)** Double ISH for **(C)** *galanin* and **(D)** *neurotensin* of a coronal section of the mouse brain (bregma –0.02 mm). **(E,F)** Double ISH for **(E)** *galanin* and **(F)** *neurotensin* of a coronal section of the mouse brain (bregma, –0.14 mm). **(G,H)** Double ISH for **(G)** *galanin* and **(H)** *neurotensin* of a coronal section of the mouse brain (bregma, –0.24 mm). Scale bars: 200 μm. 3v, third ventricle; ac, anterior commissure; ACN, anterior commissural nucleus; AH, anterior hypothalamus; BNST, bed nucleus of the stria terminalis; fx, fornix; LAN, lateroanterior hypothalamic nucleus LPOA, lateral preoptic area; MPOA, medial preoptic area; MPN, medial preoptic nucleus; NDB, nucleus of the diagonal band; opt, optic tract; PVPOA, periventricular preoptic area; SCN, suprachiasmatic nucleus; SI, substantia innominata; SON, supraoptic nucleus; VLPO, ventrolateral preoptic nucleus. Adapted from Tsuneoka et al. (2013).

In mammals, the MPOA is the brain region between the anterior commissure and the optic chiasm in the vicinity of the anterior third ventricle (Gurdjian, 1927; Paxinos et al., 2012; Simerly, 2015). The lateral POA (LPOA) is an area flanked by the MPOA medially, the bed nucleus of the stria terminalis (BNST) dorsally, and the substantia innominata laterally. The ventrolateral side of the LPOA is bordered by the nucleus of the diagonal band. The LPOA contains the medial forebrain bundle, and the medial border of the bundle corresponds to the medial border of the LPOA (Simerly, 2015). Several genes are differentially expressed between the MPOA and LPOA. For example, neurons expressing the neuropeptide galanin, which are involved in the regulation of sleep/wakefulness and reproductive behavior, are abundant in the MPOA but not in the LPOA (Figures 1, 2). Similarly, neurotensin-expressing neurons are abundant in the MPOA but not in the LPOA (Figures 1, 2). Estrogen receptor (ER) α and androgen receptor (AR), which play an important role in reproductive behavior, are also plentiful in the MPOA but not in the LPOA. Conversely, choline acetyltransferase (ChAT)-positive neurons are present in the LPOA but not in the MPOA (Uschakov et al., 2007). Therefore, although both LPOA and MPOA contain the region name POA, there are significant differences in their constituent cells and gene expression.

The MPOA contains several nuclei, such as the medial preoptic nucleus (MPN), median preoptic nucleus (MnPO), and posterodorsal preoptic nucleus (PD), according to regional differences in terms of neuron density and the expressions of various neuropeptides, neuropeptide receptors, and gonadal steroid receptors (Figure 1; Simerly et al., 1986, 1988, 1990; Ju and Swanson, 1989; Simerly, 2015; Tsuneoka et al., 2017b). The MPN is a cell-dense, highly conspicuous structure located in the medial MPOA. The MnPO is a dense cluster of small cells located on the dorsal midline of the anterior third ventricle. The PD is a small region containing large neurons close to the BNST, which abundantly expresses *proenkephalin* (*Penk*). The ventrolateral POA (VLPO) is a small region located on the ventrolateral margin of the MPOA, adjacent to the nucleus of the diagonal band (Simerly, 2015).

We have recently proposed additionally subdividing the MPOA apart from the established nuclei MPN, PD, and VLPO into four regions, namely, the dorsomedial part of the MPOA (dmMPOA), the central part of the MPOA (cMPOA), the ventral part of the MPOA (vMPOA), and the ventrolateral part of the MPOA (vlMPOA), based on gene expression (Tsuneoka et al., 2017b) (Figure 1). The cMPOA and vMPOA show higher expression of gonadal steroid receptors than the dmMPOA or the vlMPOA. The central part of the MPN (MPNc) has been shown to predominantly overlap with the sexually dimorphic nucleus of the POA in rats (Gorski et al., 1978; Bloch and Gorski, 1988), which can be identified as a dense cluster of calbindin-positive cells in rats and mice (Sickel and McCarthy, 2000; Orikasa and Sakuma, 2010; Jahan et al., 2015; Tsuneoka et al., 2017a) or by the expression of *Moxd1* in mice (Tsuneoka et al., 2017a).

The lateral subdivision of the MPN (MPNl) has a cluster of neurotensin-positive cells (Simerly et al., 1986; Tsuneoka et al., 2013). The anterior commissural nucleus (ACN) is characterized by a population of oxytocinergic neurons in the dorsal MPOA. In the mouse ACN, there are also many *thyrotropin-releasing hormone* (*TRH*)-positive neurons (Biag et al., 2012; Tsuneoka et al., 2017b). The MPNm is divided into the anterior part (MPNma), which contains a cluster of *Penk*-expressing cells, and the posterior part (MPNmp), which has a high density of *cocaine- and amphetamine-regulated transcript* (*Cart*)-expressing cells. The most posterior part of the MPNl (MPNp) was different from the main part of the MPNl in the densities of *neurotensin*-, *Penk*-, *prodynorphin* (*Pdyn*)-, and *tachykinin 1* (*Tac1*)-positive cells. Thus, as summarized in Figure 1D, the MPOA is not randomly populated with neurons that show different gene expressions but is composed of subregions that have characteristic gene expression patterns.

Recent single-cell RNA-seq analysis of the mouse MPOA demonstrated that the messenger RNA (mRNA) expression of neuropeptides such as *neurotensin*, *galanin*, *Tac1*, *Tac2*, *Penk*, *Pdyn*, *Cart*, and *Trh* contributed to the clustering of 23 excitatory subpopulations and 43 inhibitory subpopulations (Moffitt et al., 2018). In addition to single-cell RNA-seq, multiplexed error-robust fluorescent *in situ* hybridization (MERFISH), which visualizes hundreds of mRNAs at single-cell resolution with position information (Moffitt et al., 2016; Shah et al., 2016), showed that the locations of 30% of cell clusters matched each of the MPOA subnuclei (Moffitt et al., 2018). In other words, each MPOA subnucleus contains cell cluster(s) that represent and are limited to the subnucleus; in addition, there are also cell clusters that are broadly distributed across the MPOA.

Reflecting the fact that MPOA is associated with sex-hormone-related behaviors, ER and AR are highly expressed in the MPOA (Simerly et al., 1990; Murphy and Hoffman, 2001; Merchenthaler et al., 2004; Jahan et al., 2015). At the subregion level, gonadal steroid receptors are abundantly expressed in the cMPOA, MPNvl, and MPNma (Tsuneoka et al., 2017b), which are involved in sexual, parental, and aggressive behavior (Tsuneoka et al., 2015). More than 70% of *galanin*-positive neurons show ERα and AR immunoreactivity in the cMPOA and MPN (Tsuneoka et al., 2017b). *Neurotensin*-, *Penk*-, and *Tac2*-positive cell groups also showed high proportions of ERα and AR immunoreactivity, but *Trh*-positive cells did not (Tsuneoka et al., 2017b). Through region- and neuron group-specific expression of their receptors, gonadal hormones are thought to regulate sex differences in various behaviors, including sleep.

The boundaries of the MPOA can also be defined according to its characteristic gene expression. ER and AR are abundant in the MPOA, but posterior to the anterior commissure, their expression decreases sharply and is observed in only a few dorsal hypothalamic regions. Similarly, galanin and neurotensin are abundantly expressed in the MPOA with a distribution pattern similar to that of gonadal steroid receptors, but their expression is sharply reduced posterior to the anterior commissure (Tsuneoka et al., 2017b; Figure 2). Thus, we consider the oblique plane containing the posterior end of the anterior commissure and the center of the SCN to be the caudal limit of the POA. This view is also supported by the expression region of the transcription factor Nkx2.1, which plays an important role in the development of various hypothalamic structures and is abundantly expressed in the embryonic and infant POA in mice (García-López et al., 2008; Puelles et al., 2012). Thus, the MPOA subregions and LPOA can be defined according to gene expressions.

## Diverse POA Neurons Regulate Sleep/Wakefulness

The POA has long been recognized as being crucial for sleep induction (Saper et al., 2005; Liu and Dan, 2019). In the winter of 1916, there was a sudden increase in the number of patients suffering from high fever, fatigue, double vision, sleep problems, and catatonia in Vienna. Thereafter, an epidemic of this disease recurred every winter until approximately 1926. Austrian psychiatrist von Economo noticed a certain pattern of brain damage in these patients and named it encephalitis lethargica. He further found that patients who suffered from insomnia had damage in the anterior hypothalamus, while those who suffered from somnolence had broad damage rostral to the oculomotor nucleus level (von Economo, 1930). His pioneering findings provided the first argument for the existence of sleep centers in the brain. Subsequent lesion studies added to the findings supporting a role of the POA in the regulation of sleep (Nauta, 1946; McGinty and Sterman, 1968; Szymusiak and McGinty, 1986b; Sallanon et al., 1989; John and Kumar, 1998; Lu et al., 2000). For example, lesions of the MPOA using *N*-methyl-D-aspartate (NMDA) in rats reduced deep non-rapid eye movement (NREM) and rapid eye movement (REM) sleeps, shortened NREM sleep episodes, and increased body temperature (John and Kumar, 1998).

Consistent with its role in sleep induction, unit recordings of POA neurons showed the presence of sleep-active neurons (Kaitin, 1984; Szymusiak and McGinty, 1986a; Ogawa and Kawamura, 1988; Koyama and Hayaishi, 1994; Szymusiak et al., 1998; Takahashi et al., 2009; Alam et al., 2014). For example, out of 98 neurons in the rat POA, 14 neurons were active specifically during NREM sleep, and 26 neurons were most active during REM sleep (Koyama and Hayaishi, 1994). Seventy-six percent of rat MnPO neurons were sleep active, and most of them showed a gradual increase in firing rates before sleep onset (Suntsova et al., 2002). Single-unit activity of rat MnPO neurons increased during sleep deprivation but decreased during recovery sleep (Alam et al., 2014), suggesting that the activity of sleep-active POA neurons is closely related to NREM sleep delta power generation. In mice, among 872 single units in the POA and adjacent region, 552 were sleep active (Takahashi et al., 2009), and 60% of sleep-active neurons were active during both NREM and REM sleep and resting during wakefulness. Sleep-active neurons were broadly distributed within the MPOA and LPOA, and different types of sleep-active neurons, such as NREM sleep-specific, REM sleep-specific, and NREM/REM sleep-specific neurons, were intermingled in the POA. Importantly, wake-active neurons were also found in the MPOA and LPOA and were more restricted to the middle and ventral POA than sleep-active neurons (Takahashi et al., 2009). Wake-active neurons ceased firing immediately before sleep onset and started firing around 0.5 s before the onset of wakefulness. Thus, the POA contains different sets of neurons that are most active during each vigilance state.

Visualization of Fos protein as a marker of active cells showed that Fos-positive neurons during sleep and after sleep deprivation were found abundantly and broadly in the MnPO and LPOA (Cirelli et al., 1995; Gong et al., 2000; Semba et al., 2001), which is consistent with a broad distribution of sleep-active neurons in the POA (Takahashi et al., 2009). Fos-positive cells during recovery sleep were also broadly distributed in the lateral MPOA and the LPOA (Zhang et al., 2015). Genetic labeling of active neurons using Fos^CreER^ mice demonstrated that the LPOA was one of a few brain regions where the number of labeled neurons increased during recovery sleep compared to sleep deprivation (Zhang et al., 2015). More than 75% of sleep-active neurons in the POA were positive for glutamic acid decarboxylase (GAD), the rate-limiting enzyme in gamma aminobutyric acid (GABA) production (Gong et al., 2004). REM sleep restriction induced Fos immunoreactivity in GAD-positive neurons in the rat POA and MnPO (Gvilia et al., 2006), consistent with REM sleep-specific neurons in the POA (Takahashi et al., 2009). These findings indicate that the POA contains neurons that are active in different aspects of sleep regulation.

Optogenetic and pharmacogenetic manipulation of POA neurons directly demonstrated their role in the regulation of sleep/wakefulness (Table 1). Activation of a group of LPOA and lateral MPOA neurons using CNO/hM3Dq induced NREM sleep (Zhang et al., 2015). Surprisingly, activation of GABAergic neurons in the LPOA and lateral MPOA enhanced wakefulness (Chung et al., 2017). However, activation of GABAergic POA neurons that projected to the tuberomammillary nucleus (TMN), where wake-promoting histaminergic neurons are located, immediately enhanced NREM sleep and a markedly increased REM sleep within 1 min, whereas inhibition of those neurons suppressed NREM and REM sleep (Chung et al., 2017). In contrast to GABAergic POA neurons projecting to the TMN, optogenetic activation of glutaminergic POA neurons projecting to the TMN enhanced wakefulness (Chung et al., 2017). Optrode recording of 17 TMN-projecting POA GABAergic neurons revealed that they were sleep active, with their highest discharge rate occurring during REM sleep. Regarding the subtype of TMN-projecting GABAergic neurons, these neurons partly overlapped with cholecystokinin (CCK)-, corticotropin-releasing hormone (CRH)-, and Tac1-positive neurons. Whereas optogenetic activation of CCK- and CRH-positive neurons increased both NREM and REM sleep (Chung et al., 2017), optogenetic activation of Tac1-positive neurons increased only NREM sleep and not REM sleep (Chung et al., 2017). However, chemogenetic activation of Tac1-positive POA neurons increased wakefulness (Reitz et al., 2020).

**TABLE 1:** Preoptic area neuron subtypes that regulate physiology and behavior.

Marker	Localization^1^	Manipulation	Results	References
Adcyap1	VMPO	Activation	Decrease in body temperature	Tan et al., 2016
BDNF	VMPO	Activation	Decrease in body temperature	Tan et al., 2016
CCK	Lateral MPOA and LPOA	Activation	Increase in NREM and REM sleep	Chung et al., 2017
			Decrease in wakefulness	
		Inhibition	Decrease in NREM and REM sleep	Chung et al., 2017
			Increase in wakefulness	
CRH	Lateral MPOA and LPOA	Activation	Increase in NREM and REM sleep	Chung et al., 2017
			Decrease in wakefulness	
		Inhibition	Decrease in NREM and REM sleep	Chung et al., 2017
			Increase in wakefulness	
Esr1	MPOA	Activation	Enhanced pup retrieval	Fang et al., 2018; Wei et al., 2018
		Activation	No change in maternal nest building	Li et al., 2019
		Activation	Enhanced male-type mounting of both males and females	Wei et al., 2018
		Inhibition	Suppressed pup-directed behavior	Fang et al., 2018; Wei et al., 2018
		Inhibition	Suppressed male mounting	Wei et al., 2018
		Ablation	Suppressed pup retrieval	Wei et al., 2018
		Ablation	Suppressed male mounting	Wei et al., 2018
GAD2	Lateral MPOA and LPOA	Activation	Increase in wakefulness	Chung et al., 2017
GAD2, projection	Lateral MPOA and LPOA	Activation	Increase in NREM and REM sleep	Chung et al., 2017
to TMN			Decrease in wakefulness	
		Inhibition	Decrease in NREM and REM sleep	Chung et al., 2017
			Increase in wakefulness	
Galanin	Lateral MPOA and LPOA	Activation	Increase in wakefulness	Chung et al., 2017
		Activation	Increase in NREM sleep	Ma et al., 2019
			Increase in NERM sleep delta power	
		Activation	Decrease in body temperature	Ma et al., 2019
		Ablation	Fragmented sleep	Ma et al., 2019
			Blunted response to sleep deprivation	
		Ablation	Increase in body temperature	Ma et al., 2019
	Lateral MPOA	Activation	Increased in NREM sleep	Kroeger et al., 2018
			No change in REM sleep	
			Increase in delta power during all states	
		Activation	Decrease in body temperature	Kroeger et al., 2018
		Inhibition	Increase in wakefulness	Kroeger et al., 2018
			Decrease in NREM sleep	
			No change in REM sleep	
		Activation	Suppressed infanticide	Wu et al., 2014
			Enhanced pup grooming	
		Activation	No change in male sexual behavior	Wu et al., 2014
		Ablation	Suppressed pup retrieval	Wu et al., 2014
			Enhanced infanticide	
		Ablation	Suppressed male sexual behavior	Wu et al., 2014
Galanin, projection	MPOA	Activation	Suppressed male infanticide	Kohl et al., 2018
to PAG			Enhanced pup grooming	
		Inhibition	Suppressed pup grooming	Kohl et al., 2018
Galanin	MPOA	Activation	Enhanced motivation to interact with pups	Kohl et al., 2018
Projection to VTA		Inhibition	Suppressed motivation to interact with pups	Kohl et al., 2018
Galanin, projection	MPOA	Activation	No change in pup-directed behavior	Kohl et al., 2018
to MeA			Enhanced male–male aggression	
		Inhibition	No change in pup-directed behavior	Kohl et al., 2018
			No change in male–male aggression	
Leptin receptor	VMPO	Activation	Decrease in body temperature	Yu et al., 2016
Nos1 warm-sensitive	MnPO and MPOA	Activation	Increase in NREM sleep	Harding et al., 2018
		Activation	Decrease in body temperature	Harding et al., 2018
Opn5	VMPO	Activation	Decrease in body temperature	Zhang et al., 2020
		Inhibition	Increase in body temperature	Zhang et al., 2020
Pdyn	Lateral MPOA and LPOA	Activation	Increase in NREM sleep	Chung et al., 2017
Tac1	Lateral MPOA and LPOA	Activation	Increase in NREM sleep	Chung et al., 2017
		Activation	Increase in wakefulness	Reitz et al., 2020
		Inhibition	Decrease in NREM and REM sleep	Chung et al., 2017
		Inhibition	No change in wakefulness	Reitz et al., 2020
Trpm2	MPOA	Activation	Decrease in body temperature	Song et al., 2016
		Inhibition	Increase in body temperature	Song et al., 2016
Vgat	MnPO	Activation	Increase in NREM sleep	Vanini et al., 2020
			Decrease in REM sleep	
			No change in wakefulness	
		Ablation	Increase in body temperature	Machado et al., 2020
Vgat	MnPO	Activation	No change in body temperature	Vanini et al., 2020
	MPOA	Activation	Enhanced pup retrieval	Li et al., 2019
			Enhanced maternal nest building	
		Activation	No change in body temperature	Song et al., 2016
		Inhibition	Suppressed maternal nest building	Li et al., 2019
			No change in pup retrieval	
	VMPO	Activation	No change in body temperature	Yu et al., 2016
Vglut2	Lateral MPOA and LPOA	Activation	Increase in wakefulness	Chung et al., 2017
	MPOA	Activation	Decrease in body temperature	Song et al., 2016
	MnPO	Activation	Decrease in body temperature	Vanini et al., 2020
	VMPO	Activation	Decrease in body temperature	Yu et al., 2016
	VLPO	Activation	Increase in wakefulness	Vanini et al., 2020
			Decrease in NREM and REM sleep	
Vglut2, projection to PAG	Lateral MPOA and LPOA	Activation	Increase in wakefulness	Chung et al., 2017

*^1^Target sites of AAV injection according to the figures and coordinates.*

*MeA, medial amygdala; MnPO, median preoptic nucleus; MPOA, medial preoptic area; PAG, periaqueductal gray; TMN, tuberomammillary nucleus; VMPO, ventromedial preoptic area; VLPO, ventrolateral preoptic area; VTA, ventral tegmental area.*

In conclusion, we can identify diverse inhibitory neuron populations with different neurotransmitters and projection patterns, and each population is thought to regulate sleep in different ways.

Since almost all POA neurons expressing the neuropeptide galanin are GABAergic, galanin-positive POA neurons are thought to be a subgroup of POA GABAergic neurons. Optogenetic activation of galanin-positive POA neurons at high frequencies enhanced wakefulness (Chung et al., 2017). However, another study showed that optogenetic activation of galanin-positive POA neurons at a frequency close to physiological discharge rates, such as 2–5 Hz, enhanced NREM sleep but did not change the amount of REM sleep (Kroeger et al., 2018). Accordingly, optogenetic inhibition of galanin neurons in the LPOA and lateral MPOA decreased NREM sleep but did not alter the REM sleep amount (Kroeger et al., 2018). Activation of galanin-positive neurons increased the number of sleep episodes but did not change episode length, suggesting a role of galanin-positive POA neurons in the initiation of NREM sleep episodes rather than their maintenance. In addition, sustained activation of galanin neurons in the POA using pharmacogenetic tools resulted in a mild increase in NREM sleep, a decrease in REM sleep, and prominent hypothermia (Kroeger et al., 2018). Inhibition of galanin-positive POA neurons by GABAergic neurons of the ventral lateral hypothalamic area (LHA) induced wakefulness (Venner et al., 2019), which supports a sleep-inducing role of galanin-positive POA neurons.

Importantly, when galanin-positive POA neurons were photoactivated, the electroencephalogram (EEG) showed high-amplitude slow waves entrained to photoactivation and presented an increase in NREM sleep delta power (Kroeger et al., 2018), which is often used as an indicator of sleep need (Franken et al., 2001). Ablation of galanin neurons in the LPOA and lateral MPOA (defined according to their coordinates) weakened the response to 5 h sleep deprivation; mice with ablated galanin neurons showed markedly diminished increase in sleep time and a blunted increase in delta power during recovery NREM sleep (Ma et al., 2019). These findings suggest that galanin neurons in the POA play a role in the homeostatic regulation of sleep.

Galanin-expressing neurons in the POA may also be implicated in sleep in humans and fish. The number of galaninergic neurons in the human intermediate nucleus, equivalent to the rodent POA, was correlated with sleep fragmentation in older individuals with and without Alzheimer’s disease (Lim et al., 2014). Galanin-expressing POA neurons were active during recovery sleep in zebrafish. Additionally, galanin expression increased during wakefulness and sleep deprivation in zebrafish (Reichert et al., 2019). Thus, POA neurons expressing galanin may play a conserved role in sleep promotion in diverse animal species.

## Preoptic Area Fiber Connections for Sleep/Wakefulness

Microinjection of AAV-EF1α-DIO-ChR2-mCherry in the LPOA of galanin-Cre mice visualizes axon terminals in the dorsomedial nucleus (DMH), LHA, TMN, pedunculopontine tegmental nucleus, medial parabrachial nucleus, locus coeruleus, ventrolateral and lateral periaqueductal gray matter, lateral pontine tegmentum, and raphe pallidus but none in the median or MPOA (Kroeger et al., 2018). Regarding the functional connections of sleep-active neurons in the POA, neurons that express Fos during sleep and project to the paraventricular nucleus or LHA are distributed in the LPOA and MPOA (Uschakov et al., 2006). GABAergic neurons in the POA project to the POA, TMN, ventral tegmental area (VTA), locus coeruleus, orexin neurons/LHA, and laterodorsal tegmentum (Saito et al., 2013), which mainly promote wakefulness. As expected, optogenetic stimulation of GABAergic neurons in the POA inhibited orexin neuron activity in brain slices (Saito et al., 2013). Similarly, MnPO neurons send their axons to the whole MPOA and LPOA, the dorsal raphe, the locus coeruleus, and orexin neurons in the LHA (Uschakov et al., 2007). Optogenetic activation of the axons of POA GABAergic neurons in the TMN increased NREM sleep (Chung et al., 2017). In contrast to GABAergic neurons, optogenetic activation of glutamatergic POA neurons projecting to the TMN enhanced wakefulness (Chung et al., 2017).

As for the neurons upstream of sleep-regulating POA neurons, monosynaptic input to TMN-projecting LPOA neurons was found in the hypothalamus and amygdala (Chung et al., 2017). DMH neurons send their fibers to galanin-expressing GABAergic neurons in the POA (Chen et al., 2018). Optogenetic activation of these DMH neurons enhanced NREM sleep and suppressed REM sleep. Thus, there are mutual functional connections between POA GABAergic neurons and DMH neurons. In conclusion, since sleep-regulating neurons are intermingled in the POA, it is necessary to divide POA neurons into subgroups according to their afferent and efferent connections in addition to gene expression to understand how POA neurons regulate sleep/wake behavior.

## Sleep-Inducing Neurons Are not Restricted to the VLPO

**FIGURE 3:**
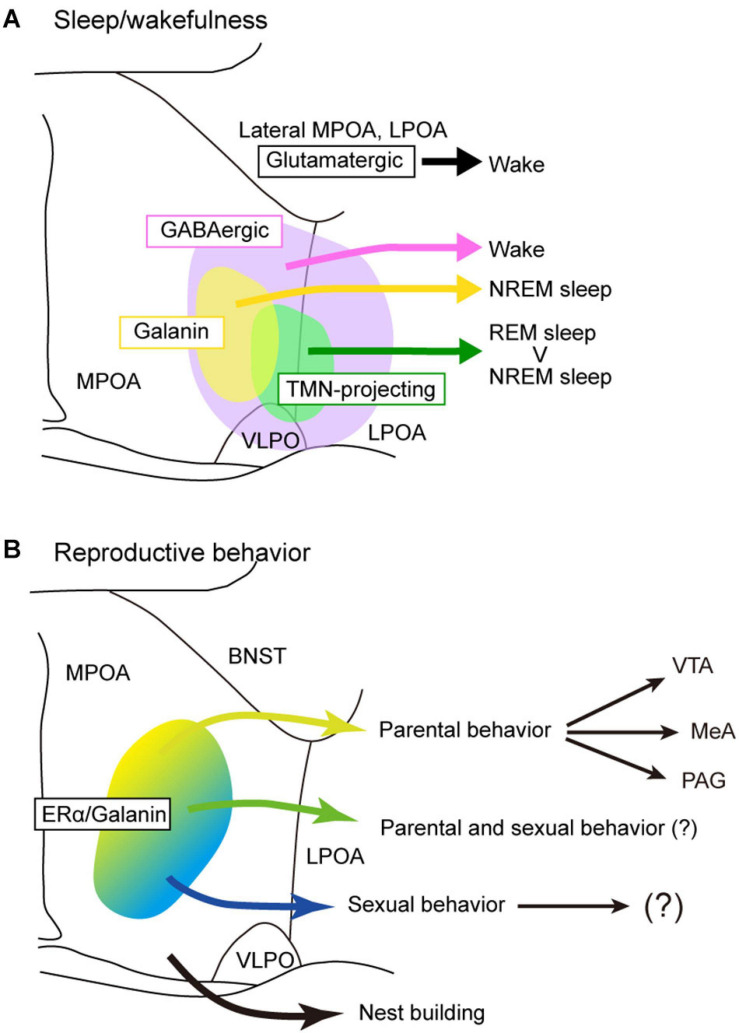
Models of POA neuron groups that regulate sleep and reproductive behavior. **(A)** Different subgroups of POA GABAergic neurons control sleep and wakefulness differently. TMN-projecting GABAergic neurons promote NREM sleep and strongly REM sleep. Galanin-positive POA neurons that are mainly GABAergic generally promote NREM sleep. POA glutamatergic neurons promote wakefulness. **(B)** Estrogen receptor α and/or galanin-positive neurons in the MPOA regulate parental behavior and male sexual behavior. Neurons involving nest building behavior was not estrogen receptor α positive. BNST, bed nucleus of the stria terminalis; LPOA, lateral preoptic area; MeA, medial amygdala; MPOA, medial preoptic area; PAG, periaqueductal gray; TMN, tuberomammillary nucleus; VLPO, ventrolateral preoptic nucleus; VTA, ventral tegmental area.

As discussed above, sleep-active or sleep-inducing neurons are broadly distributed in the lateral MPOA and LPOA (Figure 3A), which extends the idea that the VLPO is a sleep center (Saper et al., 2005; Saper and Fuller, 2017). Neurons that were Fos positive after sleep deprivation were found broadly in the medial and lateral POA (Cirelli et al., 1995; Gong et al., 2000; Semba et al., 2001). TMN-projecting GABAergic neurons are also broadly distributed in the lateral POA (Chung et al., 2017). AAV-based gene expression cannot be localized but rather tends to be widespread, and its distribution varies widely among injected mice (Kroeger et al., 2018). Therefore, optogenetic and chemogenetic activation of the LPOA regions led to broadly increased Fos-positive cells in both the MPOA and LPOA (Kroeger et al., 2018). CCK-, CRH-, and Tac1-positive neurons that enhance sleep are also broadly distributed across the MPOA and LPOA (Chung et al., 2017). Although GABA and galanin are sometimes used as markers for the VLPO (Sherin et al., 1998; Gaus et al., 2002), GABAergic neurons are distributed throughout the POA, and galanin is expressed abundantly in the medial MPOA but only sparsely in the VLPO regions (Figure 2). At the caudal end of the POA, galanin expression is sharply reduced and is restricted to the supraoptic nucleus (SON), which is caudally adjacent to the VLPO and induces sleep (Jiang-Xie et al., 2019). We have examined the expression of many genes and proteins in the POA but have not found any suitable marker for the VLPO. Since gene expression cannot clearly define the VLPO, we can identify the VLPO only according to its brain atlas coordinates. The problem is that these coordinates are not completely consistent between brain atlases. Neurons regulating sleep are not only found in the VLPO but throughout POA with diverse groups that can enhance or suppress wake, NREM sleep, or REM sleep, indicating that the POA is more than just a sleep-inducing center.

## Parental Behavior

In addition to sleep, galanin-expression POA neurons are involved in parental behavior (Wu et al., 2014; Kohl et al., 2018). Before the identification of galanin-expressing neurons as major players in parental behaviors, it had been known that the MPOA was most critical for parental motivation, especially related to pup retrieval behavior and inhibition of aggression toward pups, known as infanticide (Febo, 2011; Pereira and Morrell, 2011; Stolzenberg and Numan, 2011).

Pup retrieval is a parental behavior in which a parent picks a pup up in the mouth and carries it to the nest in response to chemical signals and ultrasonic vocalization of the pup (Okabe et al., 2010; Isogai et al., 2018). Whereas virgin female mice retrieve pups, virgin male mice often display aggression toward pups or infanticide. However, once a virgin male mouse mates and cohabitates with a female mouse, the male will show pup retrieval behavior. In other words, female mice always show pup retrieval, whereas male mice switch from infanticide to pup retrieval in a sexual experience-dependent manner.

In rats, the dorsolateral MPOA is considered to be more important in pup retrieval behavior than the ventral or medial MPOA (Jacobson et al., 1980; Numan et al., 1990). The distribution of Fos-expressing neurons differs among MPOA subregions during maternal behavior toward pups (Numan and Numan, 1994; Lin et al., 1998; Li et al., 1999; Sheehan et al., 2000; Mathieson et al., 2002). In mice, a high density of Fos expression during parental behavior was observed in the ACN, cMPOA, and vMPOA regardless of the reproductive condition of the mice, such as virgin females, parturient females, postpartum dams, and father mice (Tsuneoka et al., 2013, 2015). Lesion ablating cMPOA neurons completely abolished pup retrieval in male mice and surprisingly led virgin and parous females to conduct infanticide (Tsuneoka et al., 2013, 2015). Fos-expressing cells in the cMPOA during parental behavior are galanin-positive neurons in both females and males (Tsuneoka et al., 2013; Wu et al., 2014). Ablation of galanin-positive POA neurons enhanced infanticide (Wu et al., 2014). These results indicate that cMPOA suppresses aggression toward pups in both sexes, possibly through the activation of galanin-positive neurons.

Estrogen and prolactin regulate maternal behavior toward pups. Pharmacological blockade of estrogen in the MPOA and small interfering RNA (siRNA) silencing of Esr1 encoding ERα suppressed maternal behaviors in mice (Ribeiro et al., 2012; Catanese and Vandenberg, 2017). Acute deletion of prolactin receptors in the mouse MPOA completely abolished pup retrieval (Brown et al., 2017). Activation of Esr1-positive MPOA neurons promoted pup retrieval and ablation of Esr1-positive cells suppressed pup retrieval (Fang et al., 2018; Wei et al., 2018). Similarly, activation of galanin-positive MPOA neurons enhanced pup grooming and suppressed infanticide (Wu et al., 2014). Since most galanin-positive neurons express ERα, MPOA neurons expressing both ERα and galanin control parental behavior under the influence of estrogen (Figure 3B). In addition to estrogen, oxytocin also regulates maternal behavior. The upregulation of oxytocin signaling in the mouse MPOA promoted experience-induced maternal motivation (Okabe et al., 2017). MERFISH showed that neuron groups expressing oxytocin receptor are specifically active during pup-directed aggression (Moffitt et al., 2018). These cell populations are distinct from parental behavior-specific cells expressing calcitonin receptor and bombesin receptor (*Brs3*). However, it should be noted that the analysis included the BNST subregions in addition to the MPOA (Moffitt et al., 2018) because the BNST is also involved in parental behavior (Tsuneoka et al., 2015).

Reciprocal connections between the MPOA and medial amygdala (MeA) are important for parental behavior. Galanin neurons in the MPOA send their projections to the MeA and are activated during parenting regardless of the type of parental behavior (Kohl et al., 2018). Optogenetic activation of the MPOA galanin neuron fibers in the MeA inhibited aggression toward pups in virgin male mice, although it did not affect parental behavior (Kohl et al., 2018). MPOA-projecting MeA neurons were activated during parenting behavior, especially in father mice (Kohl et al., 2018). In female mice, optogenetic stimulation of GABAergic neurons in the MeA promoted pup grooming but not pup retrieval or crouching (Chen et al., 2019). Because almost all MPOA galanin neurons are inhibitory, they may regulate interaction with pups by inhibiting negative olfactory stimuli encoded in the MeA (Kohl et al., 2018).

Projections from the MPOA to the VTA are thought to be important for maternal motivation in rats (Numan and Smith, 1984; Hansen et al., 1991; Stack et al., 2002; Numan et al., 2005). Similarly, Esr1-positive neurons in the mouse MPOA send strong inhibitory input to non-dopaminergic neurons in the VTA, and this inhibitory input promotes maternal pup retrieval through disinhibition of dopaminergic neurons (Fang et al., 2018). Since optogenetic manipulation of the axons of MPOA galaninergic neurons in the VTA did not change parental pup retrieval (Kohl et al., 2018), galanin-positive MPOA neurons projecting to the VTA may not be involved in pup retrieval. These results indicate that galanin-negative, ERα-positive GABAergic neurons may be involved in pup retrieval via the VTA (Figure 3B).

## Nest Building Behavior

Many animals sleep in a certain posture in a nest. Rodents build nests for resting, sleeping, keeping warm, raising children, and hiding from enemies (Jirkof, 2014). Thus, nest building behavior is necessary for sleep and parental behavior. Lesions in the vMPOA of female mice suppressed nest building but did not affect maternal behavior toward pups (Tsuneoka et al., 2013). In contrast, cMPOA-lesioned mice showed disruption in both nest building and parental behavior (Tsuneoka et al., 2013). Activation of MPOA GABAergic neurons enhanced nest building (Li et al., 2019). Activation of agouti-related protein (AGRP) fibers from the arcuate nucleus to the MPOA markedly decreased nest building but only slightly changed pup retrieval behavior (Li et al., 2019). This suggests that different groups of neurons with specific fiber connections separately regulate nest building and retrieval of pups to the nest.

Although activation of Esr1-positive MPOA neurons enhanced maternal pup retrieval (Fang et al., 2018; Wei et al., 2018), activation of Esr1-positive MPOA neurons and local estrogen blockade by bisphenol S in the MPOA did not affect maternal nest building behavior (Catanese and Vandenberg, 2017; Li et al., 2019), suggesting that Esr1-positive MPOA neurons are not involved in nest building. Mouse build nests not only for nurturing but also for keeping warm. Mice nesting becomes inactive in warm conditions. Warm-sensitive neurons expressing pituitary adenylate cyclase-activating polypeptide (PACAP)/brain-derived neurotrophic factor (BDNF) in the MPOA drastically inhibited nest building behavior (Tan et al., 2016).

## Male Sexual Behavior

Conceptually, sexual behaviors are composed of appetitive and consummatory behaviors. In male rodents, appetitive sexual behavior consists of behavioral components that increase mating opportunities, such as approaching, pursuing, and sniffing. Consummatory sexual behavior is a sequence consisting of mounting, intromission, and ejaculation. Accumulated findings indicate a crucial role of the MPOA in consummatory sexual behavior (Hull and Dominguez, 2007, 2015). Electrical stimulation of the rat MPOA during mounting facilitated ejaculation (Malsbury, 1971). Lesions in the rat MPOA abolished consummatory sexual behavior repertoires (Bermond, 1982; Hansen et al., 1982; Arendash and Gorski, 1983). Such loss of sexual performance due to MPOA lesions was not reversed even after 8 months (Ginton and Merari, 1977), suggesting that there is no alternative brain region carrying out this function of the MPOA. Importantly, the number of neurons with enhanced firing changed throughout a series of mating behaviors (Horio et al., 1986; Shimura et al., 1994), and Fos expression in the rat MPOA was increased from mounting to intromission and then to ejaculation (Coolen et al., 1996; Veening and Coolen, 1998; Yamaguchi et al., 2018). This indicates that an increasing number of MPOA cells are activated as consummatory sexual behavior progresses.

Additionally, male pursuit of females disappeared in MPOA-lesioned rats (Paredes et al., 1993, 1998; Paredes and Baum, 1995; Kindon et al., 1996), and their partner preference changed from receptive females to stud males (Paredes and Baum, 1995; Kindon et al., 1996; Paredes et al., 1998). Therefore, MPOA neurons are thought to be involved in appetitive sexual behavior as well (Veening and Coolen, 2014). MPOA neurons also mediate experience-dependent sexual arousal. Sexual experience facilitates all components of mating behaviors, and this facilitation is thought to be mediated by experience-induced changes in MPOA neurons, such as synaptic plasticity (Jean et al., 2017), dopamine sensitivity (McHenry et al., 2012; Nutsch et al., 2016), oxytocin sensitivity (Gil et al., 2013), and neuropeptide precursor expression (Maejima et al., 2018).

Testosterone plays a crucial role in the regulation of male sexual behavior. After testosterone is aromatized into estradiol, this estradiol induces male sexual behavior via ERα (Sano et al., 2016). Esr1 knockdown in MPOA neurons by short-hair RNA (shRNA) drastically suppressed a series of male sexual behaviors and Esr1 knockdown in other Esr1-expressing sites such as the VMH and MeA and suppressed specific components of male sexual behaviors (Sano et al., 2013, 2016). *In vivo* calcium imaging revealed that Esr1-positive POA neurons were activated immediately after the initiation of any sexual behavior, and optogenetic activation of Esr1-positive neurons enhanced male sexual behavior (Wei et al., 2018). In addition, genetic ablation of Esr1-positive POA neurons suppressed male mounting behavior, and optogenetic inhibition suppressed both mounting and intromission in a stimulus-timing-dependent manner (Wei et al., 2018). Thus, estrogen derived from testosterone may activate a subgroup of POA neurons to promote male sexual behavior.

In addition to estrogen, oxytocin also regulates male sexual behavior. Male rat copulation was facilitated by microinjection of oxytocin in the MPOA and suppressed by an oxytocin receptor antagonist. Oxytocin binding in the MPOA was correlated with the sexual performance of males (Okabe et al., 2017).

It is not clear what types of MPOA neurons are involved in male sexual behaviors, but various neuropeptides and neurotransmitters such as α-MSH, dopamine, NPY, galanin, substance P, neurokinin K, opioids, orexin, and oxytocin have been demonstrated to affect male sexual behavior through MPOA neurons in rats (Argiolas and Melis, 2013). The distributions of Fos-positive neurons were different during parental behavior and male sexual behavior (Tsuneoka et al., 2015; Moffitt et al., 2018). catFISH analysis showed that only a small percentage (∼10%) of Esr1-positive neurons were activated during both parental and sexual behavior (Wei et al., 2018), suggesting that the cell populations involved in these two behaviors rarely overlap.

## Moxd1, a Novel Marker of Sexually Dimorphic Nuclei

In addition to reproductive behavior, there are also sex differences in sleep (Komiya et al., 2018). The total wake time of female mice is much longer than that of male mice (Funato et al., 2016). Although it is not clear what mechanism is responsible for the sex difference in sleep, sexually dimorphic structures in brains may be involved. To examine the role of sexual dimorphism in the brain, a good marker for sexually dimorphic nuclei (SDN) is necessary.

**FIGURE 4:**
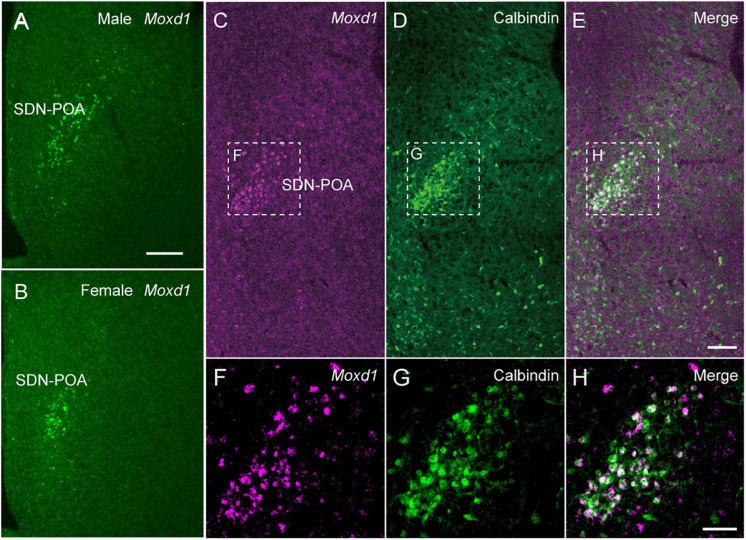
*Moxd1* mRNA as a marker for sexually dimorphic nucleus. **(A,B)** *Moxd1* mRNA expression in the sexually dimorphic nucleus of the POA (SDN-POA) of **(A)** male and **(B)** female mice. The number of *Moxd1*-positive cells of the SDN-POA are higher in males than in females. **(C,D)** The SDN-POA has cells expressing **(C)** *Moxd1* and **(D)** calbindin. **(E)** Merged images of *Moxd1* and Calbindin. **(F,G)** *Moxd1*, calbindin, merged image of dashed rectangles in panels **(C–E)**. Scale bars: **(A)** 200 μm, **(E)** 100 μm, and **(H)** 50 μm. Modified from Tsuneoka et al. (2017a).

We identified *Moxd1* as a new and specific marker gene for SDN through an *in silico* search of the Allen Gene Expression Atlas (Figure 4). *Moxd1* is expressed highly specifically in all major SDN, such as the SDN-POA, the principal nucleus of the BNST (BNSTpr), and posterodorsal part of the medial amygdala (MePD) (Tsuneoka et al., 2017a). In the MPOA, only a few *Moxd1*-positive cells exist outside the SDN-POA. *Moxd1*-positive SDN-POA cells are more numerous in male mice than in female mice (Figure 4). Subsequently, single-cell RNA-seq and MERFISH analyses have confirmed Moxd1 as a marker for a neuron subgroup in the preoptic region (Moffitt et al., 2018). Importantly, *Moxd1* expression in the SDN-POA was not affected by adult castration or ovariectomy but was affected by neonatal castration, suggesting that the expression of *Moxd1* in the SDN-POA is determined by the hormonal milieu during the perinatal period and that the expression is independent of the activating effect of gonadal steroids in adulthood. *Moxd1* encodes a monooxygenase, DBH-like 1, that is localized in the endoplasmic reticulum and is predicted to hydroxylate a hydrophobic substrate based on its amino acid sequence, which is similar to that of dopamine β-hydroxylase (Xin et al., 2004). Because the substrates of Moxd1 protein have not been identified, its biological role in sexually dimorphic neurons is not known. Although calbindin has been used as a marker of the SDN-POA, calbindin-positive cells are also distributed outside the SDN-POA (Tsuneoka et al., 2017a) (Figure 4). Thus, the higher specificity of Moxd1 allows us to visualize and manipulate SDN in order to better understand sexual differences in behavior.

## Future Directions

In summary, different approaches, including *in vivo* neurophysiology, Fos imaging, cell ablation, and optogenetic and chemogenetic manipulation, have generally shown that the POA contains a diverse group of neurons that differentially control wake, NREM sleep, and REM sleep. Detailed subgrouping of POA neurons in terms of gene markers, input/output connections, neurotransmitters, and neurophysiological properties is necessary to better understand how POA neurons regulate sleep/wakefulness.

So far, the role of the POA in sleep and other innate behaviors has been addressed separately. However, given that galanin-positive POA neurons are involved in the regulation of both sleep and parental behavior, more integrated observation will be necessary to obtain physiologically relevant insight. Since POA neurons are involved in a variety of innate behaviors associated with enhanced levels of arousal, changes in sleep/wakefulness can be secondary to increased motivation for certain behaviors. For example, whereas galanin neurons projecting to the TMN induce sleep, those projecting to the MeA promote pup-directed behavior with an increased arousal level. In this case, activation of all galanin neurons in the POA overcomes the sleep-inducing effects of a subset of galanin neurons and leads to arousal.

Although it was not the focus here, the POA serves as a hub for thermoregulation; it receives thermosensory signals from the skin and regulates downstream pathways for heat production by the brown adipose tissue and heat dissipation by the skin through vasodilation (Nakamura, 2011; Morrison et al., 2014; Harding et al., 2018; Tan and Knight, 2018; Ma et al., 2019; Morrison and Nakamura, 2019). Given that body temperature changes are associated with sleep/wake behavior, the POA may coordinately be responsible for lower body temperature during NREM sleep. The recent discovery of warm-sensing neurons in the POA expressing PACAP/Adcyap1 and BDNF demonstrated that a specific group of neurons in the POA are responsible for sensing warm environments and inducing adaptive changes that reduce heat production by brown adipose tissues and enhance heat dissipation from the skin (Tan et al., 2016). Partly overlapping neuronal populations in the midline POA have been shown to induce hypothermia (Hrvatin et al., 2020; Takahashi et al., 2020; Zhang et al., 2020).

Recent technical advances to visualize and manipulate specific groups of POA neurons will uncover how different groups of intermingled but largely separate POA neurons function in coordination as hubs for diverse behavioral modalities. How this system matured during ontogeny and evolved during phylogeny will be a future challenge. We believe that the diversity of POA reflects not only the diversity of individual behavior but also the diversity of the animal kingdom.

## Author Contributions

YT and HF contributed to the conceptualization, writing, and funding acquisition. Both authors contributed to the article and approved the submitted version.

## Conflict of Interest

The authors declare that the research was conducted in the absence of any commercial or financial relationships that could be construed as a potential conflict of interest.

## Footnotes

1 http://atlas.brain-map.org/
